# Survey of arizona emergency department infectious disease preparedness for possible ebola patients

**DOI:** 10.1186/2197-425X-3-S1-A349

**Published:** 2015-10-01

**Authors:** L DeLuca, A Pickering, Z Roward, T Durns, R Miller, D Sabb, J Cienki

**Affiliations:** Department of Emergency Medicine, University of Arizona, Tucson, United States; Department of Emergency Medicine, Jackson Memorial Health System, Miami, United States

## Introduction

The Ebola epidemic in Africa has reached 24,500 cases, and has spread to the US. The Emergency Department (ED) often serves as the first line of treatment in the US healthcare system. Arizona is the sixth largest state and consists of a few metropolitan areas and many small rural communities that rely on small EDs that lack the resources and personnel of many larger centers.

## Objectives

To assess preparedness of EDs in Arizona for a possible Ebola patient presenting via triage.

## Methods

In Fall 2014, we contacted all hospitals in Arizona with an ED listed by the Arizona Hospital and Healthcare Association, to survey their preparedness for a possible Ebola patient. Surveys were conducted through email and by phone. Each center was contacted a minimum of three times to maximize response rates.

## Results

Ten (10) EDs in Arizona completed surveys (response rate: 24%). Most were small EDs, with 80% of responses from Level III or lower trauma centers and 60% with annual ED volumes of < 40,000. 50% of the hospitals were in rural locations.

60% of the EDs had ≤ 1 isolation beds and 70% had ≤ 1 negative pressure rooms.

30% of EDs had no decontamination procedures in place and 60% stated they would use regular cleaning staff in ED rooms. 30% had no procedure for contaminated waste disposal.

90% of hospitals had created Ebola protocols within October and November of 2014. Of these, 56% (5/9) had no clear procedures for exposure of other patients, and only 40% had a protocol for exposed healthcare staff. All EDs reported Ebola training within several months of the survey.

Overall, merely 10% felt very prepared to manage a potential Ebola patient. 60% felt somewhat prepared, 20% felt they needed more time or resources to fully prepare, and 10% felt unsure whether their ED was prepared for an Ebola patient.

Most EDs were small, rural centers. Most created Ebola policies in October and November of 2014 and these were not comprehensive. Many hospitals requested clarification of national protocols, standardization of education, and access to Personal Protective Equipment (PPE) for the management of these patients.

## Conclusions

AZ EDs have begun training in Ebola protocols, but these do not appear to be comprehensive and multiple simultaneous patients would easily exceed their surge capacity. This is compounded by a lack of the nationally regulated information, resources, and equipment needed for ED preparation.

## References

[1] “2014 Ebola Outbreak in West Africa - Case Counts.” Centers for Disease Control and Prevention. Centers for Disease Control and Prevention. Web. 11 Feb. 2015.

[2] Cahen C (2015). Toward a New Nursing Identity: Organizing in the Face of Ebola.

